# The Feasibility of Remote Visual-World Eye-Tracking With Young Children

**DOI:** 10.1162/opmi.a.16

**Published:** 2025-07-26

**Authors:** Zoe Ovans, Meli René Ayala, Rhosean Asmah, Anqi Hu, Monique Montoute, Amanda Owen Van Horne, Zhenghan Qi, Giovanna Morini, Yi Ting Huang

**Affiliations:** Department of Psychology, University of Pennsylvania, Philadelphia, PA, USA; Department of Linguistics and Cognitive Science, University of Delaware, Newark, DE, USA; Department of Hearing and Speech Sciences, University of Maryland, College Park, College Park, MD, USA; Department of Communication Sciences and Disorders, Northeastern University, Boston, MA, USA

**Keywords:** eye-tracking, children, language processing, virtual research, web-camera

## Abstract

Visual-world eye-tracking has long been a useful tool for measuring young children’s real-time interpretation of words and sentences. Recently, researchers have extended this method to virtual platforms to reduce equipment costs and recruit more diverse participants. However, there is currently limited guidance on best practices, which require individual researchers to invent their own methodologies and may prevent broader adoption. Here, we present three broad approaches for implementing nine remote visual-world eye-tracking studies, and show that this method is highly feasible for assessing fine-grained language processing across populations of varying ages, clinical statuses, and socioeconomic status backgrounds. We outline strategic methods for conducting this research effectively, including strategies for experimental design, data collection, and data analysis given the variable conditions outside of a lab setting. We adopt four criteria for evaluating success for this method: 1) Minimal subject attrition relative to in-person studies, 2) Minimal track loss relative to conventional eye-tracking, 3) Conceptual replication of previous findings, and 4) Evidence of broadening participation. These case studies provide a thorough guide to future researchers looking to conduct remote eye-tracking research with developmental populations. Ultimately, we conclude that visual-world eye-tracking using internet-based methods is feasible for research with young children and may provide a relatively inexpensive option that can reach a broader, more diverse set of participants.

## INTRODUCTION

Visual-world eye-tracking has been a cornerstone tool of research in psycholinguistics for several decades, providing a well-established method for measuring young children’s real-time interpretation of words and sentences as they unfold (Blomquist et al., 2021; Borovsky et al., 2012; Contemori et al., 2018; Huang & Snedeker, 2009; Snedeker & Trueswell, 2004; Trueswell et al., 1999; Weighall, 2008, inter alia). While this technique has employed a variety of different methods, perhaps the most common implementation is to display stimuli on a screen that is in a fixed position relative to an eye-tracking computer, while participants are free to move their heads and shift their gaze within a small radius. This set-up relies on a light source (typically near-infrared light, at approximately 780–880 nm) to allow an eye-tracking camera to see the participant’s pupil, iris, and a reflection of the cornea. From this set-up, the position and direction of the participant’s eye is calculated. Various forms of this automated eye-tracking set-up may be head-mounted, in which the eye-tracking computer is in a fixed position relative to the participant’s head, or remote, in which another cue (often a high-contrast sticker on the participant’s forehead) is used to infer head position.

Visual-world eye-tracking in this way generally requires data to be collected in-person, using expensive, highly specialized, eye-tracking equipment. During the Covid-19 pandemic, many research groups either ceased data collection or developed new methods for running participants online. However, implementing virtual versions of visual-world eye-tracking to investigate children’s language processing required solving a myriad of complex challenges related to the timing of multi-channel stimuli (presenting visual pictures, auditory sentences), and precise measurement of eye movements from attention-limited, not co-present, highly mobile participants. At the time, there was limited guidance about viable approaches to solving these hurdles. Yet, necessity is the mother of invention, and the year-long suspension of in-person research has yielded invaluable lessons. The goal of this paper is to outline several viable methods for internet-based visual-world eye-tracking with children, and to discuss both sources of data loss using this new method, as well as best practices for future research. While other guides offer general advice about virtual research with children (Bogat et al., 2023; Chuey et al., 2021; Hutto et al., 2023; Kominsky et al., 2021; Lourenco & Tasimi, 2020; Sheskin et al., 2020; Shore et al., 2023) or specific replications on particular platforms (Kandel & Snedeker, 2025; Morini & Blair, 2021; Raz et al., 2024; Scott et al., 2017; Steffan et al., 2024), we hope that focusing on children’s language processing provides practical and actionable steps for flexible implementation of a difficult technique across a range of research questions and populations.

Eye-tracking methods have been a staple for studying language development because it is generally easier for children to move their eyes compared to pressing buttons or responding verbally. For children as young as six months old, meaningful eye-movements can be launched to stimuli in under a half second (Aslin & McMurray, 2004; Golinkoff et al., 2013; Irving et al., 2011; Snedeker & Huang, 2015; Trueswell et al., 1999), which makes them a useful measure of children’s reaction to linguistic input that occurs at similar speeds (Chermak & Schneiderman, 1985). Since eye-movements are sensitive to unconscious processes (Spering et al., 2011), they are also useful for uncovering children’s interpretations in naturalistic settings, without them having to understand the bounds of a particular task. However, a chief limitation of this method has been that it requires specialized hardware, namely a high-definition camera or an eye-tracker of the sort built by Applied Science Laboratories, SensoMotoric Instruments, SR Research, or Tobii Technology. This means that participants generally must be tested in a lab setting to get high-quality data. While some eye-tracker models have portable versions that can be connected to a laptop for testing outside of the lab (e.g., Tobii Pro Nano, Eyelink Portable Duo), utilizing this equipment still requires the experimenter to be physically co-present with participants.

Nevertheless, in-person data collection can be challenging for a number of reasons. In the wake of the covid-19 pandemic, wariness of in-person interaction has increased, while working parents’ time has been limited and their overall stress levels have risen (Adams et al., 2021; Dawes et al., 2021; Huang & Oppenheimer, 2021; Shum et al., 2023). Likewise, climate change has increased the occurrence of weather-related disasters due to hurricanes and wildfires, which can lead to unpredictable, sustained disruption of in-person human-subjects research that impact labs across the US (Carlin et al., 2017). Coupled with these factors, there has been a reported decrease in public enthusiasm for participating in in-person research (Cardel et al., 2020; Gwizdala et al., 2022; Hiebert et al., 2023), which parallels long-standing sentiments across underserved and understudied populations that are often excluded from research in psychology and health-related fields (Arunachalam & Huang, 2024; Levin et al., 2021; Luo et al., 2019; Prather et al., 2022). Together, this contributes to enduring challenges in recruiting a diversity of participants to address pressing gaps in knowledge. These issues are often compounded for developmental researchers recruiting narrow age ranges or specific populations (Doebel & Frank, 2024; Lourenco & Tasimi, 2020; Nielsen et al., 2017).

When in-person testing is not feasible, researchers may seek alternative methods such as running the studies on internet-based platforms. Irrespective of methods, online testing offers benefits for certain study designs, such as short experiments where the travel time exceeds the study length (Kominsky et al., 2021). Such studies are the norm in developmental research, where the number of trials is determined by the attention span of the average participant, but they make the cost-to-benefit ratio less favorable for caregivers who may judge the experience to be not worth the drive. Likewise, online testing may provide flexibility for longitudinal studies that require families to commit to participating at multiple and specific time points over the course of many months or years. Finally, this approach can provide a unique opportunity to reach specific groups who may be difficult for researchers to access in person (Hiebert et al., 2023; Lourenco & Tasimi, 2020). For example, it can enable researchers who have limited access in the local context to recruit across wider geographic regions. This strategy may be particularly beneficial for studying learners of under-resourced languages, minority dialects, and/or clinical populations that are geographically dispersed. More broadly, online testing offers a compelling avenue for increasing participant diversity in terms of age, gender, location, and socioeconomic status background. While many researchers bemoan the science built from W.E.I.R.D. participants (White, Educated, Industrialized, Rich, & Democratic), our collective ability to reach outside of the convenience sample requires strategic shifts towards embedding new technology for data collection within specific frameworks of asking and answering research questions.

Virtual data collection is not without its pitfalls. The choice of presentation and coding software can be daunting, as there are no clear standard methods for this in the field presently. Specifically, a potential drawback to online testing is the lack of standard controls over the experimental environment. Since researchers are not co-present, they may have a harder time keeping participants on-task or monitoring their performance during the study. There will be variability in participants’ testing environments introduced by the level of background noise or distractions, lighting conditions, and computer screen size and speakers. Particularly relevant for online eye-tracking research, participants will vary in the sampling rates and locations of their cameras. Variable internet connections can also introduce lag, which can be detrimental to studies that rely on a tight time course of data presentation and recording. Finally, conducting remote visual-world eye-tracking experiments requires a decision to be made about whether to hand-code gaze data from participant videos (Kandel & Snedeker, 2025; Slim et al., 2024; Snedeker & Trueswell, 2004) or use automated gaze-detection software (Webgazer: Papoutsaki et al., 2016; iCatcher: Erel et al., 2022; OpenFace: Baltrusaitis et al., 2018; PsychoPy/Pavlovia: Peirce et al., 2019). The studies described here chose the former, and best practices for hand-coding will be described.

The present paper navigates these challenges across wide-ranging studies and demonstrates that remote visual-world eye-tracking is a feasible method for assessing language processing across a variety of developmental populations. By highlighting a research domain that requires high temporal resolution, our goal is to articulate the challenges that virtual testing introduces to this line of work, and provide a menu of solutions that researchers can choose from based on their research questions. We outline case studies of three methodological strategies that our respective labs have successfully implemented across nine studies (Table 1). Unlike self-administered platforms (e.g., LookIt; Scott et al., 2017), our studies are entirely synchronous with caregivers and researchers, enabling greater scaffolding for families, more flexibility to adapt procedures in real time, and potentially broader participation of ages, populations, and (dis)abilities. For example, autistic children and those with Developmental Language Disorder have more variable support needs compared to typically developing peers, so protocols often include more trials and frequent breaks to estimate participant-level abilities. The Methods section describes the protocols developed by each lab, and organizes content based on their relations to Study creation (inputs to each platform and steps for programming), Data collection (session set-up and directions to participants), and Output (file format and next steps for data analysis). The Results section evaluates data quality for all studies based on data in regard to participant attrition (common sources of data loss at the participant level), trial-level data loss (excluded trials, frames, and frame rates), conceptual replication (sensitivity to attested patterns), and broadening participation (demographic diversity of participants).

**Table 1.** Description of task and general question for each study

**Study**	**Lab**	**Age range in years**	**Population**	**Number of participants recruited**	**Task**
*Lab1Study1*	Huang	4 to 6.5	TD	107	Syntactic parsing
*Lab1Study2*	Huang	4 to 8	Low and high SES	57	Syntactic parsing
*Lab1Study3*	Huang	4 to 10	DLD	137	Syntactic parsing
*Lab2Study1*	Qi	5 to 8.5	TD	54	Discourse comprehension with face referents
*Lab2Study2*	Qi	5 to 8.5	ASD	66	Discourse comprehension with face referents
*Lab3Study1*	Morini	1.75 to 4.17	TD	91	Word learning
*Lab3Study2*	Morini	1.75 to 2.5	TD	79	Word learning
*Lab3Study3*	Morini	2 to 2.92	TD	84	Word recognition
*Lab3Study4*	Morini	3.92 to 4.17	TD	12	Word learning
*Lab3Study1 (in-person version)*	Morini	1.75 to 4.17	TD	50	Word learning

TD = typically developing; SES = Socioeconomic status; DLD = Developmental Language Disorder; ASD = Autism spectrum disorder.

## METHODS

This section presents case studies from three developmental language labs across the Northeast U.S.: Huang lab (Lab 1: located at University of Maryland College Park), Qi lab (Lab 2: located at Northeastern University) and Morini lab (Lab 3: located at University of Delaware), and for each, describes the methods used in study creation, data collection, and data analysis. Each lab chose online testing platforms and data analysis tools to fit the unique designs required by their research questions and existing procedures within their labs. Studies in Lab 1 were investigating sentence processing and syntactic parsing, studies in Lab 2 were investigating discourse processing, and studies in Lab 3 were investigating word learning and recognition. Table 1 provides a study-specific breakdown. In total, 689 children ranging from 21 months to 10 years old were recruited across 9 online studies. They represented typically developing (TD) populations as well as children from two clinical populations, specifically autism spectrum disorder (ASD) and Developmental Language Disorder (DLD). Also, by recruiting from both existing research databases and community partnerships, our participants represented the full spectrum of socioeconomic status (SES) backgrounds, from less than $15K to greater than $200K annual household income.

**Figure 1.**
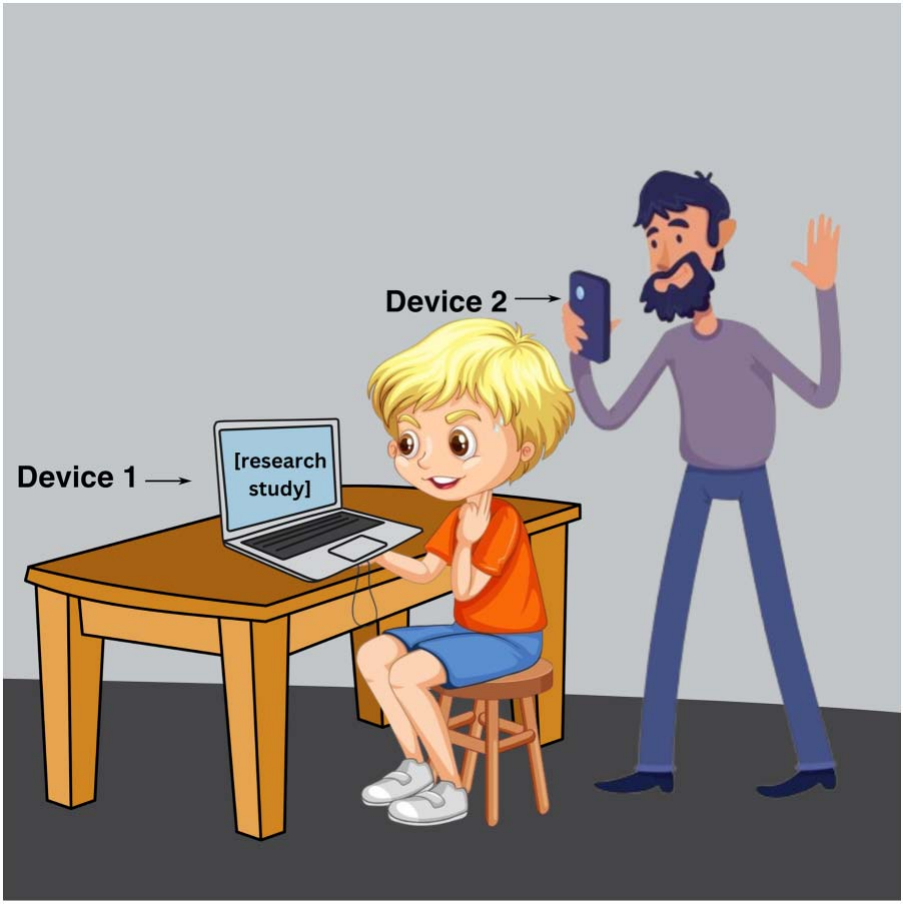
Set up for a sample study. Device 1 presents the experiment. Device 2 records child and display.

All studies required access to high-speed internet, but varied in additional hardware prerequisites. For Lab 1, families were required to have one primary (e.g., laptop, desktop) and one secondary device (e.g., iPad, iPhone), and both with functional web cameras. For Lab 2, families were asked to prepare a laptop or desktop computer that had functional web cameras and Google Chrome downloaded as a browser. For Lab 3, families needed a webcam-enabled device with at least a 12-inch screen, and access to the Camera/QuickTime apps (e.g., PCs, MacBooks). For details, see the Data Collection sections in each case study. Figure 1 illustrates the experimental set up for a sample study, including the relative position of the stimuli screen, camera(s), caregiver, and child participant. During the study, caregivers for all studies were asked to find a quiet room and avoid distractions during the appointment (e.g., turning off the TV or music). Experimenters monitored noise levels during the session and when coding videos after sessions. Since equipment variability, background noise, and home distraction impacted data quality, the Results section will evaluate how remote visual-world protocols impacted participant- and trial-level attrition. Since hardware and space requirements impose potential barriers to participation for families with fewer resources (e.g., lower-SES families), the Results section will also evaluate our premise that this paradigm is useful for moving beyond the convenience sample.

### Case Study #1

#### Study Creation.

Lab 1’s studies were run online using PCIbex (https://www.pcibex.net), an internet-based experiment hosting platform (Zehr & Schwarz, 2018). Images, audio, and video files were hosted on a separate server. Trial-level variables (e.g., condition, counterbalance list) were manipulated and recorded using a PennController script. Button press accuracy and reaction times were logged. While PCIbex does have an eye-tracker tool, studies from Lab 1 did not make use of it. Instead, they used the platform’s video recorder tool to take short videos of participants at key moments in the experiments, resulting in one video for each trial in the study. These videos were coded to begin concurrently with critical sentence audio files. Importantly, this meant that later analysis could measure word onset times in these audio files for timing analyses.

#### Data Collection.

For all the Lab 1 studies, experimenters first met participants and the caregiver on Zoom to briefly explain how the study would work. The experimenters asked that they have two devices available: a primary device that was stationary and had a webcam (e.g., a laptop, a desktop, or a Chromebook, but not an iPad) and the Google Chrome browser, as well as a secondary device that had the Zoom app and a camera (e.g., a phone, tablet, or another laptop). Caregivers were asked to place the child in front of the primary device and to position the secondary device so that experimenters were able to see both the child and the screen of the primary device. This set-up was intended to mimic the experimenter’s position in a lab study as closely as possible: the child participant in front of the eye-tracking device with the experimenter nearby, able to see and intervene or instruct if necessary. Generally, this two-device set-up meant that the child was sitting in front of the webcam-enabled computer while their caregiver was behind them and a bit to one side using the back camera through their phone’s Zoom app.

During the study, caregivers were first guided through introductory slides and an audio check for their primary device. Next, a video feed from their webcam was shown so that participants could check their lighting and the positioning of the child participant. This feed appeared on their primary device (e.g., laptop), and provided caregivers with the view of their child from the web camera’s perspective to ensure they were appropriately centered, at a good distance, and fully visible with adequate lighting. To confirm, caregivers used the camera from their secondary device (e.g., iPhone) to show the experimenters this view. Since studies were set up such that participants would be interacting with their computer screen at a normal distance, further calibration was not needed. To ensure a given participant’s videos were not reversed by their webcam software, some participants first were shown two trials on which they had to both look at an object and state whether it was on their right or left. If participants’ looks were not in the expected direction, their camera feed was assumed to be reversed. These trials also served as a calibration for coders to see clear looks to the left and right of the screen. If necessary, caregivers were instructed to resize images to fit the device’s screen.

Before the start of the study, experimenters explained the procedures to the child and gave them the opportunity to practice the task in order to ensure they understood the study. Experimenters were well trained in troubleshooting issues that might arise during the study to ensure consistency across sessions and to guard against a sample consisting only of the most technologically literate families. Most issues related to technology occurred before the experimental trials began and were resolved by switching browsers. For experiments that required children to point to objects on the screen, experimenters coded children’s actions from their Zoom vantage point during the study. At the conclusion of the experiment, experimenters and caregivers stayed on Zoom to ensure data were saved, or caregivers were given instructions for how to download and send results in the event that upload failed.

#### Output.

Participant selection data and trial-level metadata (e.g., trial number, block number, sentence, target response, etc.) were logged via PCIbex. Participant videos were uploaded on a separate server concurrently at the conclusion of each study session. Since PCIbex automatically segmented videos by trials, the onset of visual and auditory stimulus presentations was fully determined when videos were downloaded from servers for coding. Videos were converted to .mp4 format. Occasionally, participants’ computers incorrectly recorded video frame rates, and videos were down sampled using HandBrake (https://handbrake.fr/). Trial-level videos were coded using one of two video-annotation programs. VCode was initially used (Hagedorn et al., 2008), but it is no longer supported and does not run on newer Mac computers. Lab 1 therefore switched to coding in Datavyu (https://datavyu.org/; Datavyu Team, 2014). In both platforms, coders upload each trial-level video, scrub through videos frame-by-frame, and annotate for eye-movement changes (e.g., a switch to upper-left, lower-right, center, offscreen, or track loss etc.). For Lab 1, Studies 2 and 3 coded 5 areas-of-interest (top-left, top-right, bottom-left, bottom-right, center) while Study 1 coded 4 (top-left, top-right, bottom-center, true-center). Coders were instructed to place a new code at the point at which a participant’s eyes start to look in a new direction, rather than the point at which a participant’s eyes land in a new direction. Twenty-five percent of the trials were checked by a second coder who confirmed the direction of fixation, and disagreements between two coders were resolved by a third coder. Across studies, point-to-point coding agreement was minimally 90%, and Cohen’s Kappa was minimally 80%. See Snedeker and Trueswell (2004) for comparable methods for coding and analyzing frame-by-frame eye movements.

### Case Study #2

#### Study Creation.

The Lab 2 studies were programmed primarily in Gorilla (www.gorilla.sc), a web-based platform that can be used to build and run experiments (Anwyl-Irvine et al., 2020). Experimental stimuli—videos, audio, images—were uploaded to the platform and each of their experiments was programmed to allow for the desired degree of randomization or counterbalancing on both a between-block and within-block level. During trials within the experiment, Gorilla records behavioral data (e.g., participant’s selected response) as well as experimental metadata (e.g., experimental stage, trial type, trial onset time, desired answer, participant accuracy, etc). Caregivers were notified before the live session to use a webcam-enabled laptop computer or desktop that had Google Chrome downloaded as a browser. The Zoom meeting commenced with a detailed introduction of the study and parameters for participation, including ideal positioning of the child to support minimal movements and facial visibility throughout the study. Experimenters then asked caregivers to specify the device in use and open a Google Chrome browser. Participants then shared their screen, and received the link to the task on their end, to ensure their eye gazes were captured by their own webcam.

#### Data Collection.

Data for eye-tracking were collected in three ways: 1) Gorilla’s built-in eye tracker function utilizing webgazer.js (Papoutsaki et al., 2016), which has questionable temporal precision for child language research (Kandel & Snedeker, 2025; Steffan et al., 2024); 2) Gorilla’s built-in video recording system, which segmented videos by trial; 3) Zoom’s recording system, which recorded participants in a continuous stream. Generally, all three methods were simultaneously used to allow data redundancy and backups. However, if Zoom and Gorilla could not record videos simultaneously (usually on PC computers), experimenters prioritized the Gorilla video recording. If the given device (usually a PC computer) could not register the webcam within Gorilla, then Zoom would be the only means for video recording. Experimenters assisted with the progression and display of the stimuli via Zoom’s remote control feature. All videos were displayed in full-screen mode on participants’ screens.

Experimenters met participants and their caregivers on Zoom to introduce them to the general research focus, expectations of participation, and the series of activities for that session. They addressed this overview while sharing a slide deck that included examples of sitting in a comfortable location with a solid background and the ideal position for eye-tracking. These slides included screenshots of the relevant activities and upcoming tasks (e.g., collecting puzzle pieces to indicate the completion of a block and optional break). Before beginning the study, experimenters asked caregivers whether they were using a PC or Mac, and whether they had Google Chrome on their computer. They prompted caregivers to open a new Google Chrome window and share their screen, after which the experimenters shared the experiment link to the experiment via the Zoom chat. During the study, participants were given opportunities for calibration and volume checks to ensure they were comfortable and clear on the instructions, and to facilitate pristine data collection as much as possible. To ensure accuracy of manual eye gaze coding, short videos of a spinning star first on the left and then on the right side of the screen were added during each calibration phase to get a clear left-right distinction. Coders were instructed to review the calibration videos before coding and refer to the calibration videos for ambiguous frames during coding.

#### Output.

Lab 2 collected eye movement data through both Zoom and Gorilla recordings. On Zoom, the speaker view, screen view, gallery view, and combined view were recorded throughout the experiment, whether the screen was shared on the experimenter’s end or the participants’ end. Experimenters ensured that participants’ faces when captured in frame were always seen on either the speaker view or the gallery view. Participants’ audio was recorded via Zoom when their audio was shared. On Gorilla, recorded videos were zoomed-in on the participant’s face through Gorilla’s video recording function, and also captured participants’ audio. On Zoom, all these steps were recorded in one video per participant. Experimenters downloaded the Zoom recordings from the Zoom cloud and Gorilla recordings from the Gorilla server. Files were then organized and renamed using a lab-created python script that also automatically mirrored the eye-tracking video and compiled coding results (Kandel & Snedeker, 2025).

To identify the trial onsets in the recording, research assistants first manually identified the trial onsets using the screen recordings and compiled the onset timestamps for each trial and participant. With these timestamps, they then used Python to trim, save, and name the corresponding face recordings for each trial and participant. On Gorilla, each experimental trial was recorded separately, so no additional segmentation was needed. During the coding process, coders watched the trial-by-trial videos frame-by-frame, entered the eye gaze (Left vs. Right vs. Away, etc.), and added notes in the terminal command prompt. A suggested onset and offset corresponding to the actual onset and offset of the stimuli was also shown to the coder for each video. Coders used the audio from the videos to correctly locate the onset and offset while considering the suggested onset and offset timestamps. Each video was coded by two coders who watched and coded the video for left and right looks independently. Their data were compared to flag any trials where discrepancies between coders were greater than 15 frames (half a second). Any trials with discrepancies past the 15-frame cutoff were independently coded by a third coder. Data was reconciled and agreement was found between any two of the three coders. The results of the hand coding were compiled into a single file with video names, frame numbers, and directions of eye gaze.

### Case Study #3

#### Study Creation.

For experiments run in Lab 3, participants were shown a video that contained all trials and attention getters, and different versions of the video were created to preserve counterbalancing. The videos contained images of objects (either familiar or novel objects depending on the study), as well as audio files. They were created using video-editing software (e.g., FinalCut/ShotCut), and uploaded to a private account in Vimeo, a video hosting website. URL links to each video were generated and provided to families at the time that the study took place. Video recordings of participants’ looking behaviors during the study were recorded locally (on the family’s computer) using a native video-recording application (e.g., PhotoBooth or QuickTime for Macs and Camera app for PCs). Recording videos locally avoided lags in the video that would affect later coding. Additionally, a back-up recording was created via Zoom by the experimenter. During the appointment, caregivers were asked to (i) start recording the session, (ii) set the stimulus video to full-screen, (iii) hit “play,” and close their eyes for the duration of the video. Videos lasted between 6-10 minutes, depending on the study.

#### Data Collection.

Caregivers were asked to find a quiet room in the home and to limit distractions during the appointment. A detailed testing protocol with step-by-step instructions to guide the appointment, as well as verbal scripts that explained procedures to the families was implemented during every testing session. Additionally, experimenters received training on how to use Zoom and how to troubleshoot issues that may arise during the appointments across different operating systems. Together, these elements helped ensure that there was consistency across appointments, and made it possible to run appointments with families with varying levels of technical expertise. A back-up experimenter was always present in case there were internet connectivity issues with the lead tester.

At the beginning of the virtual appointment light, camera, and audio checks were completed. As part of this step, experimenters provided a link to a 30-second video clip, in which the background color changed from black to white every 5 seconds, allowing experimenters to see if the changes in brightness were detectable via the webcam. This brightness contrast is critical and would be later used to segment the start and end times of trials during offline coding. If the contrast was not noticeable, the experimenter would ask the caregiver to adjust the lighting (e.g., close/open the curtains in the room, turn on/off a lamp), and the process would repeat until the contrast was detected. To test the audio, the video included music that was presented at the same intensity level as the auditory stimuli that would be included during the experimental task. If the sound was not an appropriate volume, caregivers were instructed to adjust the volume on their computer, until the music was heard at a comfortable listening level. Once it was time to start the study (after all checks were completed), experimenters turned off their cameras, the link to the study video was provided in the Zoom chat box, and caregivers started recording the session.

#### Output.

Once the stimuli video finished, experimenters guided caregivers through steps for uploading the video of the testing sessions that they had generated using a secure file-transfer link. This was a .mov or .mp4 file that was recorded by the camera app on the family’s computer. Experimenters remained on the Zoom call until the video had been successfully uploaded (approximately 3–5 minutes). Participant videos were coded offline on a frame-by-frame basis by two trained coders using Datavyu coding software. They indicated where a trial begins and ends by looking at changes in lighting contrast across frames, and marking the start of the trial when they saw the child’s face illuminated and marking the end of the trial when the screen goes dark (i.e., the child’s face is no longer illuminated). Next, trained coders marked left and right looks within each trial, and their codes were compared. Trials where disagreement was greater than half a second were independently coded by a third coder. Data was reconciled and agreement was found between any two of the three coders for each trial. In order for a trial to be included in the final analysis, participants needed to have looked at one of the objects on the screen for a minimum of 500 milliseconds.

## RESULTS

Across the nine studies, 689 participants were recruited, and 521 participants were tested. To evaluate the success of the multiple instantiations of remote visual-world eye-tracking, we calculated metrics of data retention and data loss aggregated across all studies.^1^ We adopted four criteria for evaluating success for this method: 1) Minimal subject attrition relative to in-person studies, 2) Minimal track loss relative to conventional eye-tracking, 3) Conceptual replication of previous findings, and 4) Evidence of broadening participation. To preview our results, we find that participant cancellations are higher with remote visual-world eye-tracking (due to its virtual nature), but data retention at the participant level was comparable to in-person studies. At the trial level, data loss was higher than in-person studies but well within conventional benchmarks for developmental eye-tracking studies. Among remote visual-world eye-tracking studies that were based on existing studies, all were successful in replicating previous patterns of findings. Finally, we find enormous demographic diversity across participants in our remote visual-world eye-tracking studies, and no clear evidence that technology was a sole or primary barrier to participation.

### Participant Attrition

**Figure 2.**
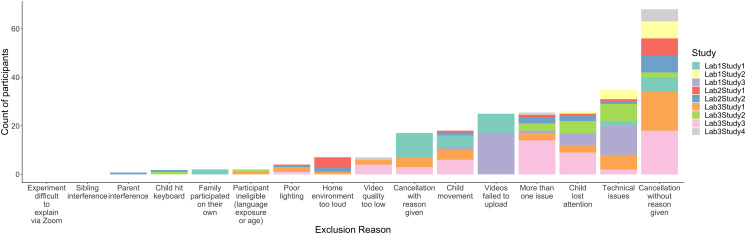
Common sources of data loss in remote visual-world eye-tracking studies.

To evaluate the causes of participant attrition, the primary units of measurement in this section focused on the level of the participant. For studies requiring multiple visits, each visit was considered a percentage of the total study. For example, for a study requiring four visits, each visit was considered .25 attendance. Figure 2 presents the number of participants who were unable to provide usable data for causes identified across the three labs. Common causes are unpacked in the sections below, and Table A1 in Appendix A provides additional description of the specific criteria used for excluding participants. To understand sources of systematic data loss, we first calculated data loss as a proportion of participants excluded for any reason divided by the total participants recruited. From a researcher’s perspective, this metric offers an overall yield rate for the total time and effort for recruitment and testing. On average, 64.0% of participants provided usable data, which is markedly lower than benchmarks from eight in-person visual-world eye-tracking studies (*M* = 85.6%; Huang & Arnold, 2016; Huang, Leech, & Rowe, 2017; Huang & Snedeker, 2009; Huang et al., 2013; Martin et al., 2022; Morini & Blair, 2021; Morini & Newman, 2021; Weng et al., 2024).

#### Source 1: Missed Appointments.

**Figure B1.**
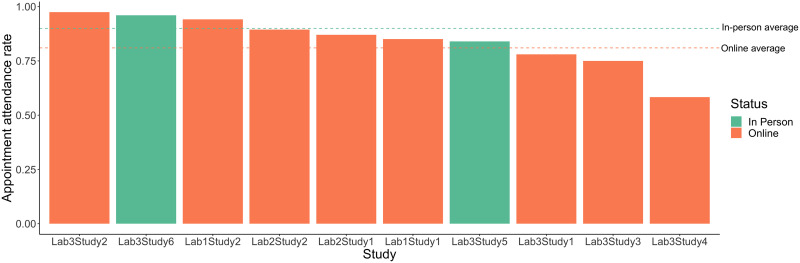
Comparison between in-person and online studies: percentage of appointments participants kept (attendance rate) Dotted lines indicate mean attendance rates for in-person and online appointments. Note that attendance rate data were not collected for Lab1Study3.

**Figure B2.**
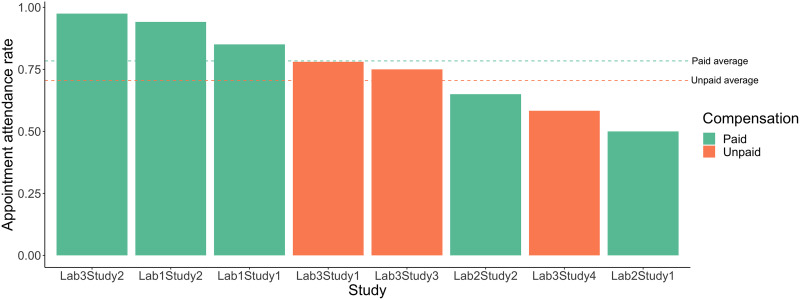
Percentage of appointments kept by compensation.

Next, we homed-in on potential causes of data loss by calculating the proportion of participants excluded for a given reason divided by those excluded for any reason. Across studies, the largest source of data loss was from participants failing to attend appointments with no reason given (28.5% of data loss overall). Sometimes families offered reasons for canceling, but this was less common (7.0%). While “no-shows” are a perennial issue for in-person testing as well, the rate was nearly double compared to similar in-lab visits. This indicates that researchers may wish to recruit more participants than they would for comparable in-person studies, though this need may vary by lab, location, and population. Figures B1 and B2 in Appendix B provide additional breakdown of attendance rate by appointment characteristics (e.g., paid vs. unpaid). Conversely, all studies were intended to be synchronous, but some families took it upon themselves to complete the study on their own. For example, Lab 1 emailed URLs to PCIbex studies to facilitate access during the appointment, but this sometimes led tech-savvy families to complete the study before the appointment (0.08% of data loss).

Another way to understand the scale of the missed-appointment issue is to benchmark it to the overall number of participants scheduled. On that basis, “no-show” accounted for 18.2% (or roughly one in five) participants scheduled. If all these participants had instead shown for their appointment, data loss for remote visual-world eye-tracking would be 82.2%. This value is strikingly similar to the average data loss for in-person visual-world studies (85.6%; Huang & Arnold, 2016; Huang, Leech, & Rowe, 2017; Huang & Snedeker, 2009; Huang et al., 2013; Martin et al., 2022; Morini & Blair, 2021; Morini & Newman, 2021; Weng et al., 2024), demonstrating that increases in “no shows” represent the largest shift in dynamics when studies move from in-person to remote. This may relate to the more impersonal nature of remote interactions compared to in-person or the perceived ease of rescheduling for the former compared to latter. Strikingly, once missed appointments are accounted for, overall participant attrition for remote visual-world eye-tracking studies is comparable to in-person eye-tracking studies.

#### Source 2: Technical Issues.

**Figure C1.**
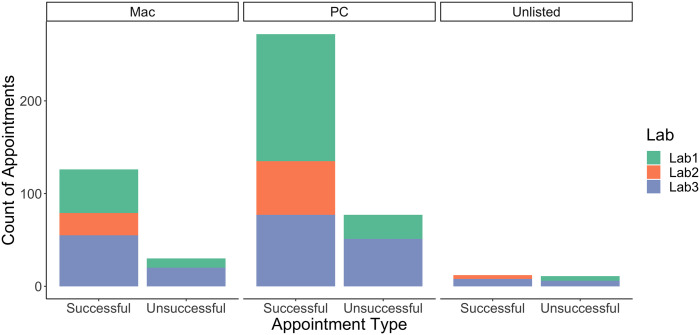
Appointment Success by Operating System (excludes no-shows and cancellations). Appointments were considered successful if that participant was not excluded from the analysis for lack of usable data.

**Figure C2.**
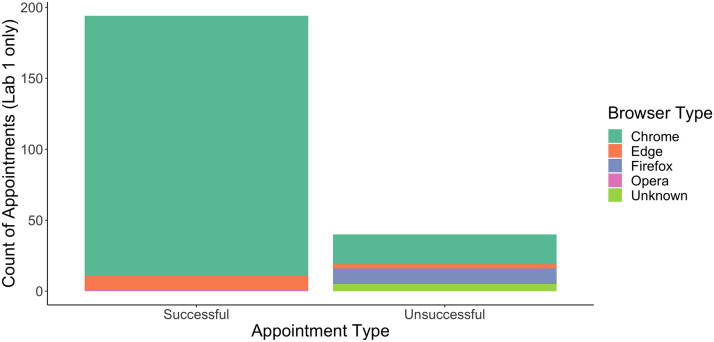
Appointment success by browser type for PCIbex studies. Appointments were considered successful if that participant was not excluded from the analysis for lack of usable data.

For participants who did attend a session, the most prominent source of data loss derived from technical issues that arose during the experiment. This category included issues such as participants’ browsers being incompatible with the experimental software, participants’ website permissions disallowing them from being recorded, or videos failing to play. These challenges arise because remote visual-world eye-tracking studies do not rely on standardized equipment and software, but instead adopt participants’ idiosyncratic technology and knowledge of how to navigate it. To analyze these issues in greater detail, we calculated data loss as a proportion of participants that were excluded for a given reason divided by tested participants. From the experimenters’ perspective, this metric provides an index of the likely challenges that could arise during a testing session, and the extent to which a given session will yield data. All told, technical issues accounted for 6.7% of participants tested. We conducted follow-up analyses to understand factors that contributed to data loss (see Figures C1 and C2 in Appendix C). While the majority of participants used PC computers running on Windows (66.0% compared to 29.5% on Macs, and 4.5% Other/Unclear), operating systems were not indicative of appointment success. In contrast, successful appointments overwhelmingly relied on Google Chrome. On average, 89.7% of Chrome appointments were successful compared to 36.6% success for all other browsers. This likely reflects study-specific guidance to use Chrome for PCIbex and Gorilla experiments since it has more versatile compatibility with video/audio recording features compared to other browsers, such as Safari and Internet Explorer/Edge. Sometimes participants were able to complete the study, but videos from their webcam failed to upload (4.8% of participants tested). This occurred less frequently than other technical issues, but often arose when families had slow internet access.

#### Source 3: Unusable Data.

**Figure 3.**
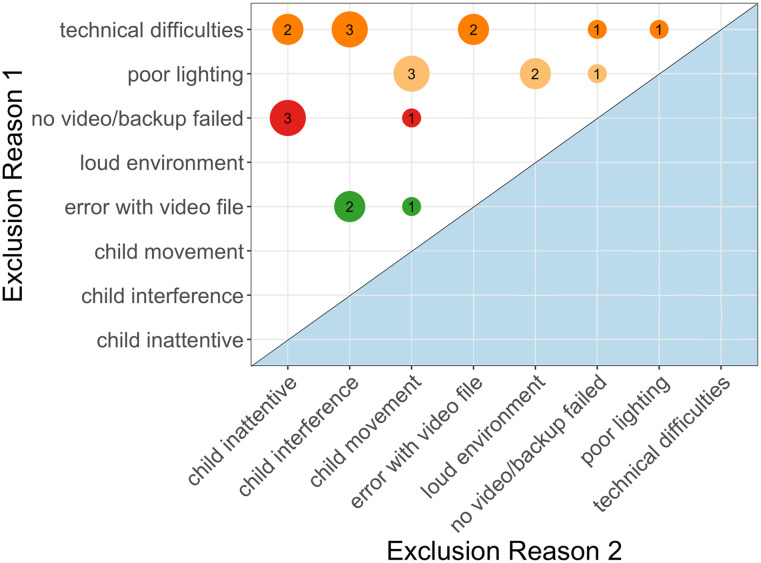
Frequency of data loss co-occurrences when multiple errors occurred. Dot size indicates frequency of co-occurrence, colors indicate levels of Exclusion Reason 1.

A third major source of data loss was participants who attended a session and completed the experiment, but their data were unable to be accurately coded because of too much movement. This occurred for 3.5% of participants tested, and was usually caused by the child ducking out of the video frame for the majority of the session. We contrast excessive movement with situations where the child completely lost attention, and stepped away from the screen altogether. This occurred for 5.0% of participants tested, and constituted the largest positioning-related issue across studies. Figure 3 illustrates that the child’s inattentiveness often co-occurred with additional factors that contributed to data loss (4.9% of tested sessions). Technical issues became more likely when children interfered with the experimental software. Similarly, child movement often co-occurred with poor lighting. These patterns highlight a central challenge in virtual studies, which is the limited influence that experimenters have to position participants and maintain engagement. This is particularly an issue with children, who have less experience with computers and are easily distracted. It also directly impacts the efficacy of remote visual-world eye-tracking, which requires sustained attention on screens and well-framed body positions to capture videos of faces. Together, this suggests the critical need to train experimenters to provide clear instructions to participants before starting the study (e.g., positioning the computer and participant, lighting and sound environment, IT checks), and to have multiple strategies to recapture the child’s attention. For example, having caregivers stay with and monitor their child for the duration of the study, introducing filler trials that are easy and fun to recapture attention, and reducing the total number of trials can be effective ways to mitigate data loss due to child movement.

Notably, a potential concern shared across all labs was the extent to which background noise and lighting conditions could be adequately controlled in home environments. This is critical for virtual eye-tracking since eye-movements are densely sampled during sentence presentation, and this requires a set-up where participants are consistently hearing audio stimuli at an audible level (e.g., away from dogs, siblings, lawn mowers, televisions), and ambient light is conducive for clear face recordings (e.g., no blurry or dark images from insufficient light or backlighting from the sun). Yet, somewhat surprisingly, neither background noise nor lighting conditions presented large issues, accounting for 1.3% and 0.8% of tested participants, respectively. Potential reasons for these low numbers are that participants were for the most part seen during the day, and caregivers during the pandemic had become familiar with helping their children frame themselves in front of a webcam during semesters of virtual schooling. It could also be the case that modern computer software often contains algorithms for filtering out background noise in their microphones, so recorded videos were quieter than ground-truth environments.

### Trial-Level Data Loss

Another way to assess data quality is to evaluate the amount of usable data that each participant contributed when they were retained in the study. For the 441 participants who provided usable data, we calculated the rates of excluded trials, track loss, and frame rates across the nine studies. Note that the threats to data quality at the trial level overlap with those at the participant level (e.g., squirmy children, distractions, poor lighting conditions, slow internet access). However, assessing impacts at the trial level provides a finer-grained evaluation of the efficacy of remote visual-world eye-tracking. Since eye-movements are highly variable behaviors to begin with, it is critical to retain many trials per subject in order to effectively estimate the influences of fixed effects. Also, since trial loss varies substantially with child attentiveness, which in turn varies with age, we report these values in aggregate and by lab.

#### Excluded Trials.

We first calculated the percentage of trials that were fully excluded from further analyses. This metric broadly captures wide-ranging causes for data loss, including experimenter error, equipment track loss, and coder disagreement, and assesses platform stability and the degree to which experimenters were well-trained to implement the study. For each study, we divided the number of excluded trials by the number of total trials. For five comparable in-person studies, rates of excluded trials are very low (*M* = 1.5%), particularly when testing school-aged children (Huang & Arnold, 2016; Huang, Leech, & Rowe, 2017; Huang & Snedeker, 2009; Huang et al., 2013; Martin et al., 2022). Across nine virtual-world eye-tracking studies, we found that overall trial loss was notably higher (*M* = 7.9%), but within conventional benchmarks for retaining participants in developmental eye-tracking studies. It also varied across labs (Lab 1: 1.1%, Lab 2: 16.3%, Lab 3: 27.2%), but this may reflect in part the higher data loss associated with testing younger participants.

#### Track Loss.

To evaluate data attrition within each trial, we calculated the percentage of frames that were coded as track loss for each study. In eye-tracking studies, track loss can arise for a variety of reasons, including participants’ blinking, looks away from the display, or research assistants’ inability to infer locations for fixations due to excessive participant movement. Hence, this metric operationalizes the degree to which tasks are potentially too difficult or boring or surrounding environments are noisy and distracting. For each trial, we divided the number of excluded frames by the number of total frames per trial, and averaged percentages of retained trials for each study. For five comparable in-person studies, track loss averaged 11.6% (Huang & Arnold, 2016; Huang, Leech, & Rowe, 2017; Huang & Snedeker, 2009; Huang et al., 2013; Martin et al., 2022), demonstrating that experimenters are able to effectively maintain child attention when they are co-present. In contrast, we found that overall track loss was 27.7% across the six remote visual-world eye-tracking studies that collected trial-level data. Notably, it was consistent across labs that collected trial-level data (Lab 1: 25.6%, Lab 3: 28.4%), but tested highly disparate ages. While this value is within conventional benchmarks for retaining trials, it suggests that the virtual methodology is associated with elevated data loss.

#### Frame Rates.

One potential concern was that remote visual-world eye-tracking may have less precision in estimating fixation durations compared to modern desktop eye-trackers, which are calibrated to sample fixation locations every 2 milliseconds. This concern is offset by the fact that saccades only occur every 300 milliseconds on average (Andersson et al., 2010; Carter & Luke, 2020; Matin et al., 1993), and frame rates are standardized to 30 frames per second for modern computer cameras. However, frame rates are important to consider when analyzing videos that draw from highly disparate software, web cameras, and internet speed (Semmelmann & Weigelt, 2018; Slim & Hartsuiker, 2023; Slim et al., 2024; Vos et al., 2022). Failing to normalize for frame rates can mean that some participants are contributing more data per time window than others.

**Figure 4.**
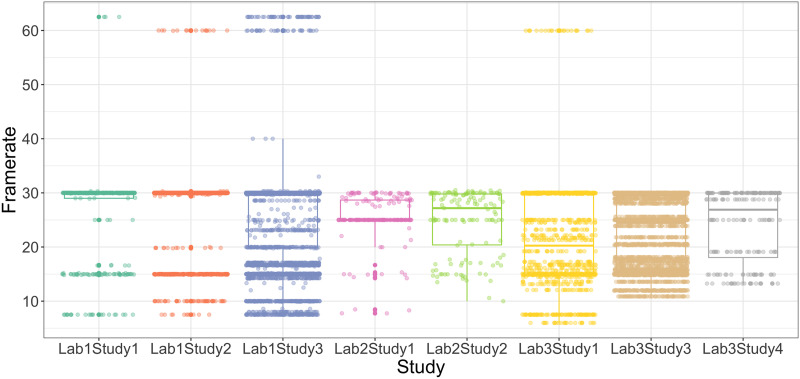
Video frame rates for all studies. Points represent individual video files. *Note*: Trial-level data for Lab3Study2 was unavailable for analysis.

To evaluate the extent to which this was an issue in our studies, Figure 4 calculated frame rates for each trial and examined their distribution across labs and studies. For Lab 1, PCIbex consistently collected videos at the standard 30 frames/second (74.2% of all videos). A smaller subset (14.8%) had a framerate of approximately 15 frames/second, and a tiny proportion (0.05%) had approximately 60 frames/second. For Lab 2, data from Gorilla were also usually 30 frames/second, with 27.0% at lower frame rates potentially due to fluctuations in participants’ internet speed. For Lab 3, local recordings were also largely done at 30 frames/second (28.1%). Even so, there was variation, with 22 trials (0.05%) sampled even below 10 frames/second. One way of normalizing across participants is to choose the modal frame rate (e.g., 30 frames/second), and systematically fill in missing data points for videos below this frame rate. This is a viable strategy for videos with frame rates high enough to capture most gaze data (i.e., above 15 frames per second), but it is ill-advised for lower-resolution videos, where one or more saccades may have occurred between frames. If participants saccade to a new area of the screen between frames, it may appear in analysis that their looks are more delayed than they are in truth. We will return to this issue in the Discussion.

### Did Remote Visual-World Eye-Tracking Studies Replicate Previous Findings?

Another way to evaluate data quality for remote visual-world eye-tracking studies is to assess the extent to which they yielded data that are sensitive to attested patterns in the literature. Given the elevated trial-level data loss associated with this method (see Trial-Level Data Loss section), this validation would be crucial for determining whether the retained trials and frames are useful for assessing fine-grained psycholinguistic phenomena. Eight out of our nine remote visual-world eye-tracking studies were based on existing protocols developed for traditional eye-tracking. One study was a direct replication of an in-person protocol (Morini & Blair, 2021). All others were conceptual replications that tested documented effects using different items (e.g., words, sentences, visual displays) or populations (e.g., ages, SES background, disability status) (Asmah et al., 2022; Hu & Qi, 2023; Ovans, 2022; Van Horne et al., 2025). While data analysis is on-going, our preliminary findings reveal strong parallelism between traditional eye-tracking and remote visual-world studies. For each lab, we briefly describe the study goals, existing evidence, and replicated patterns.

#### Lab 1.

Studies 1 and 2 investigated whether school-aged children predict likely meanings of ambiguous PP-attachment after the onset of instrument, modifier, and equibiased verbs (e.g., “Lena is going to hit the bear with the blicket. Look at the blicket.”). These studies used a traditional visual-world display, and monitored eye-movements to pictures of likely instruments and modifiers (Asmah et al., 2022; Ovans, 2022). Preliminary findings replicate well-documented patterns of incremental syntactic parsing found with video-based eye-tracking (Snedeker & Trueswell, 2004; Yacovone et al., 2021), head-mounted eye-tracking (ASL-5000 eye-tracker; Kidd et al., 2011), and desktop eye-tracking (Tobii T120 Eye Tracker; Bavin et al., 2016). Study 3 investigated whether children with DLD incrementally update role assignment for active and passive sentences after the onset of verb morphology (e.g., “The horse is brushed by the sheep”), and measured eye-movements to pictures of agent-first vs. patient-first events (Van Horne et al., 2025). Preliminary findings replicate patterns found with TD children using head-mounted eye-tracking (I-Scan Mobile Eye-Tracker; Stromswold et al., 2002) and desktop eye-tracking (Tobii X120 eye-tracker; Abbot-Smith et al., 2017).

#### Lab 2.

The design of Study 1 and 2 was motivated by the paradox where social attention on one hand guides word learning in early development (e.g., Baldwin & Moses, 2001; Tomasello et al., 2007), and on the other hand, it does not appear to be necessary to gate word learning (e.g., Akhtar et al., 2001; Foushee et al., 2023). Both studies reported here investigated how social scenes (child-directed vs. overheard) modulated children’s attention to speakers’ faces and subsequent word learning outcomes in both TD and autistic samples. This word-learning paradigm has not been used in any in-person lab visits. However, similar in-lab eye-tracking paradigms using video stimuli have shown greater fixation to speakers in interactive than non-interactive social scenes (Tobii X120 eye-tracker; Parish-Morris et al., 2019). Our preliminary analyses are based on 10 TD and 13 autistic children, and they provide similar proofs-of-concept, that is, greater looks to the speaker in the interactive child-directed context than the non-interactive overheard context (Hu & Qi, 2023).

#### Lab 3.

Study 1 and 2 investigated the extent to which toddlers learn new word-object relations better when information is provided in spoken sentences using child-directed-speech prosody, compared to songs (e.g., “Look at the doop! Do you see the doop? Where is that doop? Doop!”). These preferential looking studies measured eye-movements to the left vs. right side of the display, and directly replicate patterns found in data collected using in-person testing (preferential-looking paradigm; Morini & Blair, 2021). Likewise, Study 3 examined the extent to which background noise makes it harder for children (particularly those being raised bilingual) to recognize familiar words (e.g., “Look at the apple! Can you find the apple? Apple!”), compared to when no noise is present in the background. This study measured eye-movements to the left vs. right side of the display, and conceptually replicates previous findings collected in a lab setting with children of the same age (Morini & Newman, 2021).

### Did Remote Visual-World Eye-Tracking Studies Broaden Participation?

**Figure 5.**
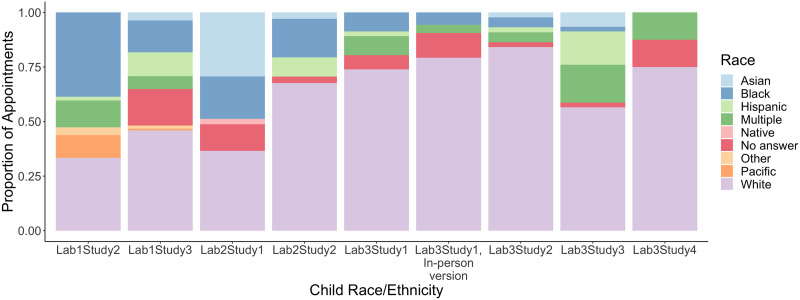
Race and ethnic backgrounds of participants by lab and study.

A chief motivation for developing robust protocols for remote visual-world eye-tracking is the potential to broaden research participation in developmental psycholinguistics, particularly for lower-incidence populations (e.g., developmental language disorder, autism, bilingualism in the US) or populations that are underrepresented in research (e.g., lower-SES families). As discussed, remote visual-world eye-tracking may increase no-shows and appointment times during data collection, and track-loss and manual coding time during data analysis. However, to the extent that it makes national recruitment feasible, the method may pave the way to pursue questions that could not otherwise be asked. To evaluate the extent to which technology helped or hindered our ability to achieve this objective, we analyzed demographic information about families that was collected from eight out of nine remote visual-world eye-tracking studies and one in-person study. Figure 5 illustrates the race/ethnic backgrounds of 413 participants for whom demographic data were obtained.

Given the range of populations that were recruited, it was not straightforward what benchmarks were appropriate for assessing efficacy. Study-by-study comparisons with in-person protocols would be difficult to interpret since differences could be affected by regional demographics of where the in-person vs. virtual studies were conducted (e.g., labs in Iowa vs. Maryland). Instead, we took the variety of our studies to be a chief strength of this project, and focused on the extent to which their demographics in aggregate successfully achieved a representative sample of children in the US, and how this changes based on whether: 1) participants contributed usable data, and 2) studies focused on underrepresented populations (see Scott & Schulz, 2017 for a similar approach). From 2020–2023 (i.e., the time period when data were collected), children under age 18 years were 49% White, 26% Hispanic, 14% Black, 5% Asian, and 6% were multiple races/ethnicities or other categories (US Census, 2020–2023). Likewise, approximately 20% of children lived below the poverty line, which is defined as making less than $30,000 annually or having an Area Deprivation Index of 80 or above (US Census, 2020–2023). With respect to disability status, approximately 3% of children were diagnosed with autism (CDC, 2025), and 7% of children were diagnosed with DLD (NIDCD, 2025).

Since participants who did and did not contribute usable eye-tracking data were recruited for the same set of studies, any obvious differences in demographic characteristics could provide hints to possible barriers to participating in remote visual-world studies. However, when we classified participants along this dimension, we did not find systematic patterns in participant characteristics. On average, the 356 participants who provided usable eye-tracking data and demographic information were 59.4% White, 5.5% Hispanic, 12.2% Black, 5.3% Asian, 1.5% Pacific islander, 0.04% Native American, and 9.2% were multiple races/ethnicities or other categories. Approximately 9.6% reported annual income below the poverty line, 6.5% were autistic, and 31.9% had a diagnosis of DLD. An additional 4.9% chose not to provide demographic data. Likewise, the smaller group of 57 participants who did not provide usable data were on average 56.9% White, 1.8% Hispanic, 19.7% Black, 7.3% Asian, and 1.1% were multiple races/ethnicities. Approximately 10.9% were autistic, and 6.3% had a diagnosis of DLD. An additional 13.3% chose not to provide demographic data. Importantly, no participant in this category lived below the poverty line, which runs counter to the hypothesis that technology requirements impose barriers to research participation.

Next, we examined the extent to which remote visual-world eye-tracking enabled recruitment of populations that traditionally underrepresented in research. In aggregate, our studies oversampled with respect to children with disabilities, since this was a central focus of two out of nine studies. Based on the demographics of participants who contributed data, our studies collectively met US benchmarks with respect to Black and Asian participants, but drastically undersampled Hispanic participants. This likely reflects the fact that two studies recruited Spanish-English bilinguals (Lab 3), but all others focused on monolinguals. Likewise, we undersampled lower-SES participants since only one study focused on SES variation. Together, this suggests that research questions play a central role in driving recruitment. To simulate participant characteristics if studies collectively did not ask questions about underrepresented populations (i.e., no bilinguals, SES variation, disabilities), we analyzed the four studies that recruited typically developing, monolingual children. This is the eligibility criteria for the “average” language development study in the literature. Relative to the overall group of participants who contributed data, this smaller group of 121 participants were on average more likely to be White (64.4%) and Asian (6.2%), and less likely to be Hispanic (0.6%), Black (6.7%), multiple races/ethnicities or other categories (8.3%), and have incomes below the poverty line (6.3%).

Together, our findings suggest that technology is not a panacea for broadening participation, but it may be a critical tool in a diverse toolkit to enable a broader range of questions to be asked, which in turn increases the collective diversity of our science. This perspective adds nuance to traditional discussions about convenience sampling, which often focus on its negative impacts on generalizing study findings to broader populations (Doebel & Frank, 2024; Lourenco & Tasimi, 2020; Nielsen et al., 2017). However, implicit in these arguments is the premise that the same research questions would be relevant across all populations. This may be true in some cases and entirely false in others. Since the lives and experiences of bilingual children, children with disabilities, and children from lower-SES backgrounds differ from their monolingual, non-disabled, higher-SES peers in notable ways, pushing the boundaries of our field’s collective knowledge will be tied with our ability to develop, validate, and adopt tools to investigate wide-ranging learners and learning contexts.

## DISCUSSION

This paper presents a proof-of-concept for implementing nine visual-world eye-tracking studies with young children using internet-based data collection strategies from three laboratories. Across studies, we found that it is possible to successfully recruit and test children, and hand-code the data using a variety of data collection and annotation platforms. We found that the largest stumbling block for remote visual-world eye-tracking was participants’ failing to attend their scheduled appointments. This problem is shared with virtual studies more broadly, but impacts eye-tracking disproportionately because of the large numbers of participants that are needed to accurately estimate eye-movements. Among sessions that occurred, the primary source of data loss arose from technical issues which occurred across platforms, operating systems, and browsers. Strategies to mitigate include providing a brief “tech-check” prior to the start of the study, and training researchers to offer clear instructions for navigating IT issues during sessions. Finally, among sessions that yield data, the primary contributor of poor data quality was mostly caused by child movement during the study. This issue is true for lab-based testing as well, but is exacerbated when experimenters are not co-present with participants. Mitigating strategies include asking participants to find a comfortable seating arrangement in a less distracting environment, including frequent breaks to avoid attention loss, and redesigning studies that are shorter in duration.

Notably, remote visual-world eye-tracking performs remarkably well with respect to data quality, particularly when appointments are synchronous and eye gaze is manually coded. While prior validations have focused mostly on adult participants (e.g., Semmelmann & Weigelt, 2018; Slim & Hartsuiker, 2023; Slim et al., 2024; Vos et al., 2022; but see Kandel & Snedeker, 2025), it was unclear the extent to which these findings would hold for children, who on average generate longer fixations, more motion artifacts, and may simply walk away when bored. Across wide-ranging ages, study questions, and populations, we found that data loss at the level of participant, trial, and frames was generally higher compared to in-person studies, but were well within conventional benchmarks for developmental eye-tracking studies. This highlights the robustness of our protocols in the face of technological and procedural challenges. Moreover, preliminary analyses indicate that all visual-world eye-tracking studies were able to directly or conceptually replicate prior findings (Asmah et al., 2022; Hu & Qi, 2023; Morini & Blair, 2021; Ovans, 2022; Van Horne et al., 2025), demonstrating that this method yields usable data for assessing fine-grained language processing in children.

Our findings suggest that researchers need not purchase specialized equipment or software to run visual-world eye-tracking experiments. However, there are lab- and study-specific factors to consider when deciding between implementations of this method. Table 2 provides a summary of the pros and cons of the three methodological strategies from the perspective of the researcher, caregiver, and eye coder. Overall, methods like PCIbex and Gorilla have the advantage of integrating stimuli presentation and data recording within a single platform. However, they require upfront costs related to programming experiments and are well suited for labs that have this in-house expertise. Conversely, the Zoom/QuickTime method leverages software that caregivers are familiar with, but requires additional steps for uploading and segmenting videos. On the testing side, PCIbex requires and Gorilla recommends using a Chrome browser. Caregivers were generally compliant and did not have trouble downloading and installing this software when needed, but it did add time to testing sessions. While it may be useful for the field to develop testing methods that are not browser-dependent, this can be practically difficult to implement as new browsers become popular, and permissions on old browsers are subject to change.

**Table 2.** Benefits and drawbacks of various methods used for researchers and caregivers

		**PCIbex (Lab 1)**	**Gorilla (Lab 2)**	**Zoom/QuickTime (Lab 3)**
**Researchers**	**Pros**	Experiments can be asynchronous	Has a GUI and pre-set study templates for common paradigms	Provides back-up recordings in case videos are not uploaded
		Support and documentation are available	Support and documentation are available	Does not require an internet connection to record
		Free and open-source	Integrates with json library, HTML, and javascript	
		Integrates with HTML and javascript		
	**Cons**	No GUI available, requires time to learn how to code experiments	Fee required for data collection	Requires a separate platform to present stimuli
**Caregivers**	**Pros**	Study is easy to launch	Caregivers only need to be present for a short time (up to 10 minutes) at the beginning of the experiment to help set up	Caregivers are familiar with Zoom, making it easy to access and use
		Experiments can be asynchronous	Video recordings are automatically uploaded through Gorilla at the end of the experiment	
	**Cons**	Loading times can be long if videos are large	Caregivers need to receive instructions synchronously from an experiment administrator and help with initial setup for web-cam recording.	Always requires caregivers to send videos manually
		Can require caregivers to download and send videos manually if they fail to upload		
**Gaze coders**	**Pros**	Auto-segments videos into trials	Auto-segments videos into trials	None
	**Cons**	Video quality can drop with internet connection	Video quality can drop with internet connection	Video quality can drop with internet connection; Videos must be manually segmented into trials

On the data-quality side, our findings demonstrate that track loss was roughly 2× higher for remote visual-world eye-tracking relative to in-person testing. While this elevated rate did not stymie our ability to replicate patterns in the literature, it raises questions of benchmarks for minimum viable track loss in remote studies. At a theoretical level, this relates to conditions for achieving adequate power to detect an effect. On average, studies with more AOIs require more within-trial saccades to accurately estimate each location, which imposes lower thresholds for track loss. Conversely, more trials and subjects generally increase sampling opportunities, which enable higher thresholds for track loss. At a practical level, however, the impacts of these variables interact directly with task performance. For example, Kandel and Snedeker (2025) compared 2- vs. 4-image displays for cohort-competition effects in 5- to 6-year-olds. While doubling the number of AOIs should theoretically decrease the effect size when the number of trials and subjects are held constant, they were in fact highly similar across display types. This underscores the importance of supplementing intuitions and simulations with empirical validations when deriving best practices. Interestingly, Kandel and Snedeker (2025) also found that manual coding of participant videos achieved effect sizes that were 4× greater than automated eye-tracking using WebGazer, suggesting that methods of inferring location may have a much larger impact on study power, over and beyond issues of track loss and sample size.

**Figure 6.**
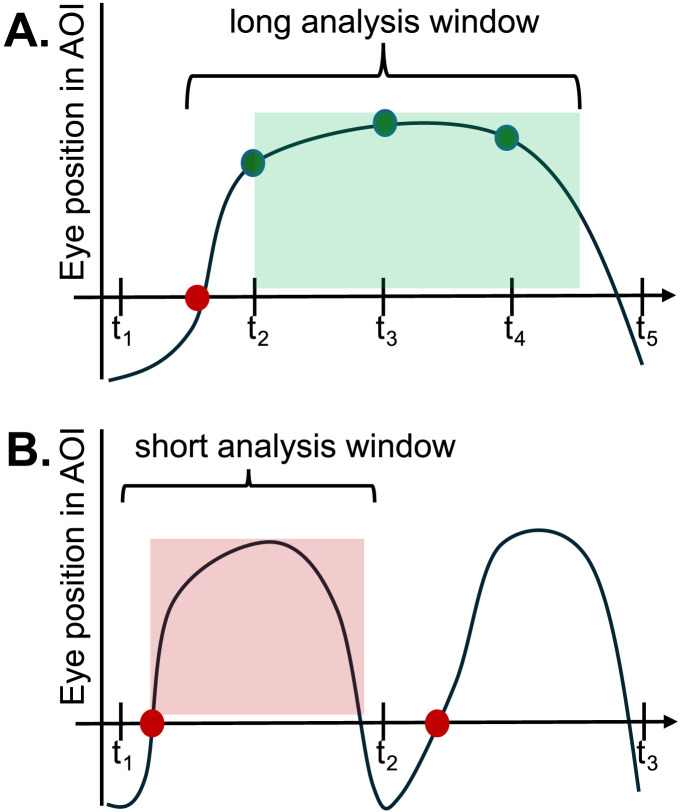
The *x*-axis shows the sampling interval of the camera. The *y*-axis shows the eye’s ground-truth position within a target AOI. (A) Shows a best-case scenario where the eye enters the AOI (red dot) and is sampled 3× by a higher-speed camera within a long analysis window (t2, t3, t4). The green box shows an extended period where stimulus-relevant fixations are detected by the camera. (B) Shows a worst-case scenario where the eye enters the AOI (red dot) and leaves before it is sampled (t2). The red box shows that with a short analysis window, relevant fixations can be missed by a lower-speed camera.

Likewise, our findings replicate the presence of substantial frame-rate variability in webcam tracking (Semmelmann & Weigelt, 2018; Slim & Hartsuiker, 2023; Slim et al., 2024; Vos et al., 2022), raising questions about minimum viable frame rates (see Andersson et al., 2010 for more discussion). This relates to a camera’s ability to detect fixations within an AOI when they exist, which is easier when phenomena unfold over a long temporal envelope (e.g., verb prediction effects last ∼800 ms) and harder when they are more fleeting (e.g., cohort effects last ∼400 ms). Moreover, fixation durations are generally longer for young children compared to adults (Helo et al., 2014), making them easier to detect at lower frame rates. Based on an analogy to bird watching, this is akin to the adequacy of slower cameras for spotting pigeons (children) compared to hummingbirds (adults). As a rule of thumb, Figure 6A shows an optimal trial where a participant’s eyes enter the AOI (red dot) and is sampled multiple times within an analysis window (t2, t3, t4 = hits). In contrast, Figure 6B shows a less ideal scenario where the eyes enter the AOI (red dot) and leave before they can be sampled by a lower-speed camera (t2 = miss). At a practical level, however, frame rates and sampling windows can interact with task performance in idiosyncratic ways. For example, Slim et al. (2024) found that effect sizes for verb prediction in adults were unexpectedly smaller compared to cohort competition, driven by participants’ tendency to maintain center fixations in the former compared to latter. Likewise, fixation durations vary substantially both between individuals (Blomquist et al., 2021; Borovsky et al., 2012; Gomes et al., 2025; Huang, Leech, & Rowe, 2017; Huang et al., 2013; Stromswold et al., 2002; Van Horne et al., 2025; Yacovone et al., 2021) and within (Huang, Newman, et al., 2017; Huang & Ovans, 2022; Martin et al., 2022), suggesting that thresholds for viable frames rates may best be determined by empirical benchmarks from prior or pilot studies.

Finally, the demographic diversity of children enrolled in remote visual-world eye-tracking studies was remarkable, and contrasts with long-standing challenges to move beyond convenience sampling in developmental research (Arunachalam & Huang, 2024; Doebel & Frank, 2024; Nielsen et al., 2017; Prather et al., 2022). While additional work is needed, we found no evidence that families who were able to provide data differ drastically from those who did not, suggesting that access to technology may not be a primary barrier to research participation (cf. Lourenco & Tasimi, 2020). Conversely, we did find that study goals (e.g., focusing on disabilities, bilinguals, SES variation) directly impacted sampling strategies and the overall demographic diversity of studies. Together, this suggests that technology alone is not a panacea for broadening participation, but is simply one of many tools for exploring the full range of questions that are relevant to the diversity of children in the US. In particular, remote visual-world eye-tracking may offer an essential method for closing gaps in recruiting lower-incidence (e.g., autism, DLD) and underrepresented populations (e.g., lower-SES families) by opening the door for national recruitment. This feasibility gap is not subtle. In the post-pandemic environment, one back of the envelope estimate suggests that recruiting a single child with DLD costs approximately $1600 and 57 hours when relying on in-person methods alone compared to $483 and 12 hours through remote methods (Hiebert et al., 2023). These recruitment costs contrast with the 5 hours it takes to manually gaze code a 72-trial study for one DLD participant. Manually coding is labor-intensive work, but it is relatively small in comparison to the investments needed to recruit lower-incidence populations.

More broadly, there remain very sensible reasons to avoid remote visual-world eye-tracking altogether and continue to run studies in well-controlled lab environments instead. Since young children can be more distractible in home environments, studies that require constant attention may not be ideal for this setting. Since remote visual-world eye-tracking relies on participants’ equipment, software, and internet, it is well suited for populations that already have access to this infrastructure or can be readily provided with such. That said, we have shown that technology barriers are surmountable, and possible avenues include loaning equipment to participants or partnering with local schools and libraries (e.g., Lab1Study2). Relatedly, additional resources for recruitment will be needed to compensate for higher attrition compared to in-person studies. One way to offset this hurdle is to incorporate collective systems of recruitment within virtual platforms such as LookIt eye-tracking within Children Helping Science (Scott et al., 2017), the online CRADLE (Collaboration for Reproducible and Distributed Large-Scale Experiments; Sheskin et al., 2020), and Psychological Science Accelerator (PSA; Moshontz et al., 2018). While such studies often adopt asynchronous protocols, this paper highlights the benefits of synchronous protocols for enabling greater scaffolding for families, more flexibility to adapt procedures in real time, and potentially broader participation of ages, populations, and (dis)abilities.

Finally, remote visual-world studies yield data that require frame-by-frame coding by humans, which requires substantial investments of time and training for labs. These procedures may eventually be replaced with automated gaze-detection software to code children’s eye-movements (e.g., Baltrusaitis et al., 2018; Erel et al., 2022; Peirce et al., 2019), and additional validations across wide-ranging studies and populations are needed. For visual-world studies, the most widely studied tool to date is Webgazer.js (Degen et al., 2021; Papoutsaki et al., 2016; Semmelmann & Weigelt, 2018; Slim & Hartsuiker, 2023; Steffan et al., 2024; Vos et al., 2022; Yang & Krajbich, 2021). However, head-to-head comparisons with manual gaze coding suggests that it is currently less accurate at detecting short and subtle effects in adults and children (Kandel & Snedeker, 2025; Slim et al., 2024). In contrast, manual coding yields data with a temporal and spatial resolution on par with lab-based, automated eye-tracking (see Did Remote Visual-World Eye-Tracking Studies Replicate Previous Findings? section; Kandel & Snedeker, 2025; Slim et al., 2024; Snedeker & Trueswell, 2004), suggesting that this is preferable for studies that prioritize these dimensions. It is worthwhile to note that time estimates for manual coding vary drastically across studies (see Appendix D). Kandel and Snedeker (2025) reported a 7-second video took roughly one minute to code for typically developing school-aged children, but we find that a 5-second video takes four minutes to code for similarly aged children with DLD, who are often restless in language tasks. Ultimately, decisions about which platform to use may depend on a study’s resources for participant recruitment vs. manual gaze coding. If the circumstances are right (e.g., plentiful population, easy-to-recruit participants, many-trial study, no societal disruptions), then in-person, automated eye-tracking may remain the most effective data-collection strategy.

In conclusion, we demonstrate that multiple instantiations of remote visual-world eye-tracking provide feasible avenues for measuring children’s language processing. While recruitment rates may need to be higher for online studies to compensate for participant drop-out, other potential pitfalls such as poor lighting or environmental noise do not present large issues in practice. Overall, we see this method as a substantial step forward in making developmental psycholinguistics and allied fields more inclusive, and ensuring that the knowledge created represents the range of language learners and users in the world and not only ones that are able to come to our labs. Having established multiple reliable methods for stimuli presentation and data collection for remote visual-world eye-tracking, future work may focus on coordinating other elements of this technique that remain bottlenecks in research. We hope that demonstrating the feasibility of this difficult technique in young children will inspire the field to pursue additional creative avenues for conducting research outside the lab.

## ACKNOWLEDGMENTS

We would like to thank the following people for helpful discussions about our eye-tracking methods: Sophie Domanski, Kathleen Oppenheimer, Megan Kanaby, Jonet Artis. We would also like to acknowledge many research assistants in each lab for quality coding (general and frame by frame) and participant scheduling: Kelly Chan, Sarah Gracia, Emily Cohen, Veronica Foster, Hannah Shapiro, Jessica Orozco-Contreras, Lucy Young, Chloë Miller, Leslie Puma, Sarah Gagné, Yuri Kim, Archana Sathiyamoorthy, Nyomi Fox, Rochelle Nelson, James Harvey, Valerie Hsieh, Aliya Yafarova, Katherine Richard, Madison Pruitt, Sarah Dombroski, Shakhlo Nematova, Abhigna Rao, Aurora Reible-Gunter, Lauren Mellor, Ben Cushman, Claudia Kurtz, Sarah Blum, Aashaka Desai, Kathryn Catalino, Katrina Conner, Mariann Angela Agapito, Shruti Shirapu, Paige Kassman, Jillian Lardiere, Talia Gillespie, Silpa Annavarapu, Jessica Price, Brianna Postorino, Dea Harjianto, Sydney Horne, Ryan Moore, Taylor Hallacy, Emily Fritzson, Kathryn Catalino, Nicole Khanutin, Jackson Xiao, Chaithra Reddy, Emily Arena, Nicole Scacco, Sophia Emery, Elizabeth Smith, Erin Felter, Kathryn Hall, Katherine Filliben, Amanda Kalil, Jessica Michels, Kate Chirinos-Cazar, Mackensie Blair, Pumpki Lei Su, Samantha Kennedy, Shreeya Parekh, Alexandra Stone, Annabelle Goetter, Brooke Barrett, Emma Frampton, Lindsay Hawtof, Morgan Smith, Stephanie Krause, Stephanie Stollar, Sydney Goldstein, Tyler Hecht, Alanis Perez, Ashanti Craig, Eden Groum, Ekaterina Novikova, Fahima Chowdhury, Johana Garcia-Mendoza, Mackenzie Popp, Madeline Austria, Wendy Sanchez-Rodriguez, Zoe Cronin, and Hannah Wissner. We would also like to acknowledge Anthony Yacovone for sharing the original version of the python script for eye-tracking data coding for Lab2 studies.

## FUNDING INFORMATION

Gorilla Research grant 2019 awarded to Anqi Hu. Lab1Study3 work was funded by NIH NIDCD 1R01DC018276-01A1 (Owen Van Horne). Lab1Study1 and Lab1Study2 were funded by NSF BCS-1844194 (Huang) and BCS-2313939 (Ovans).

## AUTHOR CONTRIBUTIONS

ZO: Conceptualization; Data curation; Formal analysis; Project administration; Visualization; Writing – original draft. MA: Data curation; Writing – review & editing. RA: Data curation; Visualization; Writing – review & editing. AH: Data curation; Writing – review & editing. MM: Data curation; Writing – review & editing. AV: Conceptualization; Writing – review & editing. ZQ: Conceptualization; Writing – review & editing. GM: Conceptualization; Writing – review & editing. YH: Conceptualization; Methodology; Supervision; Visualization; Writing – original draft; Writing – review & editing.

## DATA AVAILABILITY STATEMENT

The data and analysis materials for this manuscript are available at the following link through our OSF repository: https://osf.io/2nhsj/?view_only=9459cd7e2e9d4ea3b8f49e76f82e50f7.

## Note

^1^ All data and analysis code for this project can be found at https://osf.io/2nhsj/?view_only=9459cd7e2e9d4ea3b8f49e76f82e50f7.

## Figures and Tables

**Figure D1.**
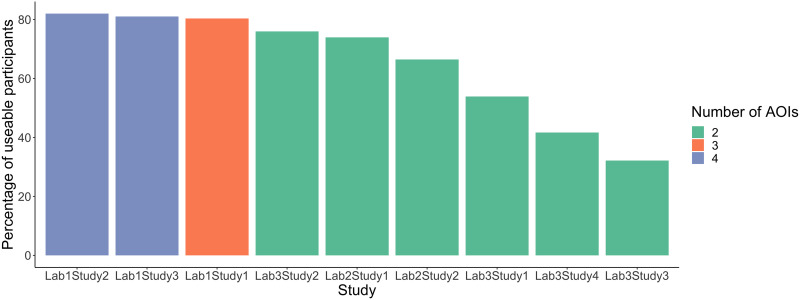
Percentage of participants included in the analysis (e.g., not dropped for track loss or inability to code) by number of areas of interest presented in the experiment.

**Figure D2.**
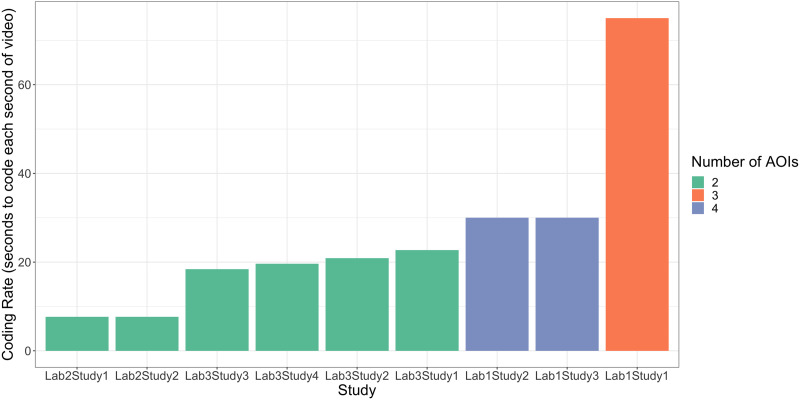
Coding rate by number of areas of interest for each study.

## APPENDIX A: COMMON SOURCES OF DATA LOSS IN VIRTUAL-WORLD EYE-TRACKING STUDIES

**Table A1.** Description of the specific criteria used for data exclusions

**Reason for exclusion**	**Detail**
Family participated on their own	Ahead of meeting with the family, the researcher sent a link to the experiment, along with instructions to wait to open it. However, the caregiver opened the link and completed the experiment with the child before meeting with the researcher.
Videos failed to upload	The child completed the experiment, but the videos collected never uploaded to the server. It is unclear why exactly: it could be due to hardware or Wi-Fi issues on the family’s side or some problem of communication between the study platform and the server where data was stored.
Cancellation with reason given	Family emailed the researchers to cancel participation in the experiment (i.e., generally due to unplanned conflict).
Cancellation without reason given	Family did not show up to the meeting and did not notify the researchers beforehand.
Technical issues	The family was unable to complete the experiment because various elements (e.g., images, sound files) didn’t load, generally due to slow internet.
Child movement	Child moved around so much during the experiment that they were out of frame for most of the resulting videos.
